# Metal-Centered Photoredox Catalysis using d^6^ Transition Metal-based Chromophores

**DOI:** 10.1021/acs.accounts.6c00326

**Published:** 2026-07-13

**Authors:** Micheal M. Alowakennu, Bekah E. Bowers, Björn Pfund, James K. McCusker

**Affiliations:** Department of Chemistry, 3078 Michigan State University, 578 South Shaw Lane, East Lansing, Michigan 48824, United States

## Abstract

Controlling excited-state reactivity in transition metal chromophores is central to light-driven chemistry, with applications spanning from solar energy conversion to photoredox catalysis. Ru­(II) and Ir­(III) chromophores currently dominate applications within this field due to their long-lived charge-transfer (CT) excited states, which support bimolecular electron transfer chemistry. However, their elemental scarcity and relative lack of selectivity for differentiating oxidative versus reductive pathways has in part motivated the exploration of alternative platforms that could, in principle, offer distinct mechanistic opportunities.

Earth-abundant, valence-isoelectronic 3d^6^ complexes of Fe­(II) and Co­(III) have attracted significant attention due to their electronic similarity to Ru­(II) and Ir­(III), suggesting the potential for complementary photophysical behavior. In contrast to their heavier congeners, however, the weaker ligand fields of first-row transition metals promote rapid relaxation from initially populated CT states to low-lying metal-centered (MC) excited states, typically of ^5^T_2_ or ^3^T_1_ character. Because these MC states involve redistribution of electron density within MC d-orbitals rather than charge separation between metal and ligand orbitals, they were historically considered poor candidates for use in photochemical transformations.

In this Account, we summarize our and others’ recent work that challenges this paradigm by establishing MC excited states in Fe­(II) and Co­(III) complexes as distinct and mechanistically powerful platforms for productive photochemistry. We begin by outlining how the nature of the reactive MC excited state can be identified and how its spin-state character impacts photochemical reactivity. This is important because MC excited states are defined by complex potential energy landscapes in which multiple competing pathways are present and can be profoundly different with regard to both spin state and equilibrium geometry. For Fe­(II) complexes, ^5^T_2_ excited states are unreactive in photoredox processes due to substantial reorganization energy and limited access to low-spin Fe­(III) products. In contrast, ^3^T_1_ excited states of Fe­(II) and Co­(III) enable productive electron-transfer pathways by minimizing spin and structural reorganization, providing access to Fe­(III) and Co­(II) products under favorable conditions. These insights establish key design principles for tuning reactivity through ligand field strength and excited-state electronic structure.

Together, these findings establish MC excited states as a fundamentally distinct platform for photoredox catalysis with advantages – including enhanced selectivity – that can be leveraged in ways difficult, if not impossible, to realize in conventional CT-based systems. More broadly, this work has allowed for the development of design rules for exploiting MC excited states of earth-abundant, first-row transition-metal complexes, presenting new opportunities in photoredox catalysis by controlling spin state, ligand field strength, and structural reorganization associated with excited-state electron transfer reactions.

## Graphical abstract

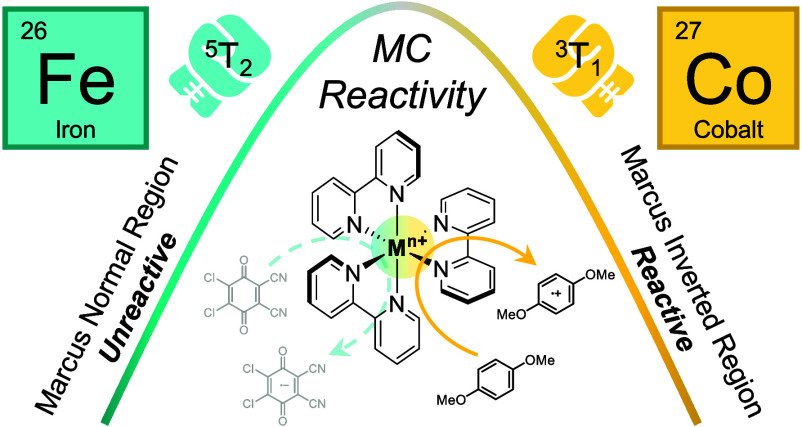

## KEY REFERENCES

-

Bowers, B. E. ; Pfund, B. ; Beissel, H. F. ; Ghosh, A. ; McCusker, J. K. Spin-State and Reorganization Energy Considerations for Metal-Centered Photoredox Catalysis. J. Am. Chem. Soc. 2025, 147, 39898–39911 41099334 10.1021/jacs.5c14935PMC12576781.^1^ This study establishes that large reorganization energies and spin restrictions render the metal-centered ^5^T_2_ excited state largely redox inert, while providing a rationale why Co­(III) complexes work and that stronger ligand fields could enhance the photoredox reactivity of the ^3^T_1_ metal-centered excited state.
-

Ghosh, A. ; Yarranton, J. T. ; McCusker, J. K. Establishing the Origin of Marcus-Inverted-Region Behaviour in the Excited-State Dynamics of Cobalt­(III) Polypyridyl Complexes. Nat. Chem. 2024, 16, 1665–1672 38965436 10.1038/s41557-024-01564-3.^2^ This paper describes the application of variable-temperature time-resolved absorption spectroscopy to a series of Co­(III) polypyridyl complexes. In conjunction with analyses of the data using Arrhenius, transition state, and Marcus theories, this study establishes that ground-state recovery dynamics of this class of chromophores occurs in the Marcus inverted region and that the photoactive state is the ^3^T_1_ state.
-

Chan, A. Y. ; Ghosh, A. ; Yarranton, J. T. ; Twilton, J. ; Jin, J. ; Arias-Rotondo, D. M. ; Sakai, H. A. ; McCusker, J. K. ; MacMillan, D. W. C. Exploiting the Marcus Inverted Region for First-Row Transition Metal-Based Photoredox Catalysis. Science 2023, 382, 191–197 37824651 10.1126/science.adj0612PMC10690870.^3^ By exploiting Marcus inverted-region behavior, this study demonstrates photocatalysis with cobalt­(III) polypyridyl complexes, where higher excited-state energies yield longer-lived and photoreactive ^3^T_1_ metal-centered states, enabling photocatalytic reactivity inaccessible to conventional Ru­(II) and Ir­(III) photocatalysts.
-

Alowakennu, M. M. ; Ghosh, A. ; McCusker, J. K. Direct Evidence for Excited Ligand Field State-Based Oxidative Photoredox Chemistry of a Cobalt­(III) Polypyridyl Photosensitizer. J. Am. Chem. Soc. 2023, 145, 20786–20791 37703518 10.1021/jacs.3c09374.^4^ This study establishes that the ^3^T_1_ metal-centered excited state of Co­(III) polypyridyl complexes can participate in bimolecular oxidative electron transfer photoreactivity and is photoredox active.
-

McCusker, J. K. Electronic Structure in the Transition Metal Block and Its Implications for Light Harvesting. Science 2019, 363, 484–488 30705184 10.1126/science.aav9104.^5^ This paper discusses the origin of the differences in electronic structure between first-row transition metal complexes and their heavier congeners, providing the conceptual framework for this entire subfield of research.

## Introduction

Photochemistry plays a central role in modern synthetic chemistry and solar energy conversion by enabling the generation of highly reactive excited states from visible light.^6–10^ Among molecular chromophores, Ru­(II) and Ir­(III) transition-metal complexes with a d^6^ valence electronic configuration have long served as benchmark photocatalysts.^11–13^ Their electronic structures provide access to long-lived charge-transfer (CT) excited states with strong visible-light absorption and tunable redox potentials, enabling a broad range of light-driven transformations.^14^ Despite their success, the scarcity and cost of ruthenium and iridium pose challenges for large-scale, sustainable applications.^5,15^ This has motivated intense efforts to develop photocatalysts based on earth-abundant first-row transition metals.^16^ In particular, as valence-isoelectronic counterparts to their second- and third-row congeners, 3d^6^ complexes of Fe­(II) and Co­(III) have attracted significant interest as earth-abundant alternatives.^17–23^ However, the smaller radial distribution of 3d orbitals induces weaker ligand fields, thereby stabilizing metal-centered (MC) excited states relative to CT states.^5^ Consequently, initially populated CT states decay within picoseconds to low-lying MC states,^24–26^ precluding their use in diffusion-based photochemistry.^27^ Over the past decade, major efforts have therefore focused on ligand design strategies that destabilize MC states and restore CT states as the lowest excited state in these systems.^28–31^ Additional approaches also seek to utilize spectroscopic techniques to inform ligand design by kinetically suppressing excited-state relaxation via modification of the reaction coordinate to introduce larger activation barriers,^32,33^ or by identifying vibrational modes involved in nonradiative decay processes and thereby restricting these motions through synthetic manipulation.^34–36^ Despite these advances, charge-transfer excited-state lifetimes in Fe­(II) and most Co­(III) complexes remain on the picosecond time scale and are therefore generally too short to support diffusion-controlled photoredox catalysis.

Rather than extending CT-state lifetimes, this Account explores an alternative paradigm that harnesses the photochemical potential of MC excited states in Fe­(II) and Co­(III) complexes. In these 3d^6^ systems, the lowest MC excited state corresponds to either a ^5^T_2_ or a ^3^T_1_ ligand-field state, which can enable reactivity distinct from, and in some cases superior to, conventional CT-state photoredox catalysis.

Because MC excited states involve redistribution of electrons among the metal-centered d-orbitals,^5^ the nature of the excited state (^5^T_2_ or ^3^T_1_) critically influences photochemical reactivity. Therefore, this Account first outlines a variable-temperature transient absorption methodology that allows for identification of these states,^2,37^ followed by an analysis of the spin requirements governing their electron-transfer reactivity.^1^ We then examine the photochemical behavior of Fe­(II) and Co­(III) complexes^1,3,4^ and conclude by outlining emerging design principles for earth-abundant photocatalysts featuring MC excited states as their lowest excited state.^1,38^

## Methodology for Determining the Nature of MC Excited States

The identity of MC excited states can be challenging to assess using conventional transient absorption spectroscopy, in part because of the variety of ligand-field excited states of differing structure and spin that a given molecule possesses coupled with the fact that the technique is not inherently spin-sensitive. Time-resolved X-ray absorption and emission spectroscopy can directly probe the spin character of MC excited states, but these methods require highly specialized and resource-intensive instrumentation.^39–45^ To address this issue, we employed variable-temperature transient absorption (VT-TA) spectroscopy to analyze the ground-state recovery (GSR) process of complexes exhibiting MC excited states.^2,37,46^

The temperature dependence of these GSR dynamics (assuming the recovery occurs subsequent to excited-state thermalization) can be analyzed using a variety of theoretical constructs, including the Arrhenius model, transition-state theory (Eyring model), and semiclassical Marcus theory.^2,32,37^ The Arrhenius model provides information on the activation energy (*E*_a_) associated with relaxation from the excited state to the ground state, as well as a frequency factor that can be viewed as the rate constant in the barrierless limit (Figure 1a, left). In contrast, transition-state theory provides deeper insight by separating the activation free energy (ΔG^‡^) for GSR into enthalpic (ΔH^‡^) and entropic (ΔS^‡^) contributions through the Eyring equation (Figure 1a, center).^47^ Finally, semiclassical Marcus theory extends transition state theory by explicitly accounting for the structural reorganization energy (λ) required to transform the excited-state geometry into that of the ground state, as well as the electronic coupling (H_ab_) between the two states,^48^ via the Landau-Zender formalism (Figure 1a, right). Although Marcus theory is most commonly associated with electron transfer, it fundamentally describes nonradiative transitions and can therefore also be applied to excited-state relaxation dynamics.^2,32,37^

**Figure 1:**
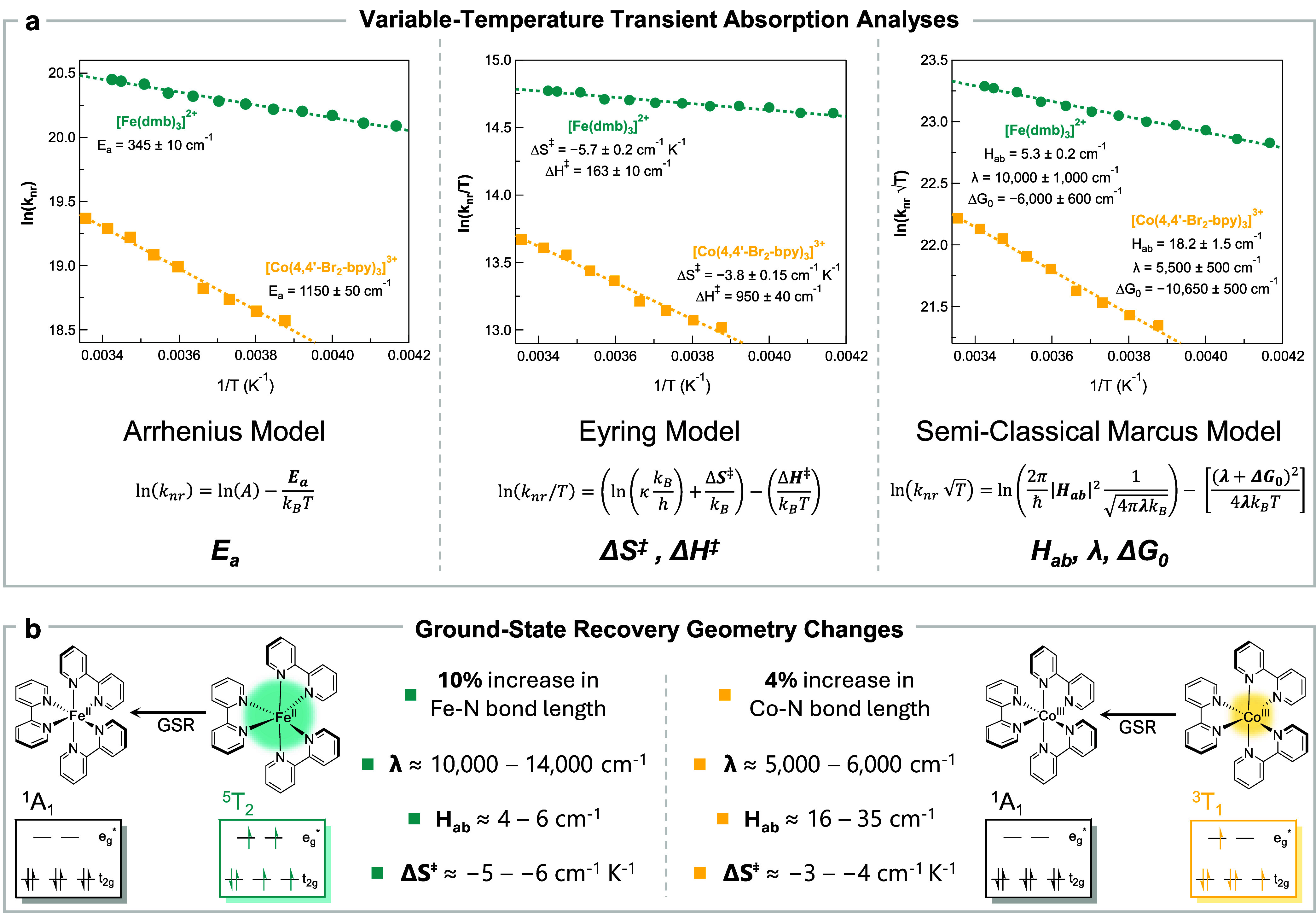
(a) Linearized Arrhenius (left), Eyring (center), and semiclassical Marcus (right) models for Fe­(II) (teal) and Co­(III) (orange) bipyridyl complexes. (b) Geometry changes during relaxation from MC excited states in Fe­(II) (left) and Co­(III) (right) polypyridyl complexes and their influence on the reorganization energy (λ), activation entropy (ΔS^‡^), and electronic coupling (H_ab_).

Eyring and Marcus analyses not only provide information on the GSR process but also allow us to determine the nature of that excited state through the lens of ΔS^‡^, λ, and H_ab_. Since the lowest energy excited state of various Fe­(II) polypyridyl complexes (e.g., [Fe­(bpy)_3_]^2+^) has been definitively established as the ^5^T_2_ state,^39,41,49^ these systems provide a useful benchmark for the interpretation of data acquired on other compounds. Therefore, we compared the parameters extracted for Co­(III) complexes and newly developed strong-field Fe­(II) systems, for which the nature of the lowest excited state was unknown, with those of Fe­(II) polypyridyl complexes.

Semiclassical Marcus theory parametrizes the GSR process in terms of ΔG_0_, λ, and H_ab_ through the linearization of the Marcus equation.^37^ Plotting the rate constant for GSR from variable-temperature time-resolved absorption measurements as ln­(*k*_nr_√T) versus 1/T yields a straight line whose slope and intercept can be correlated with the parameters of interest (Figure 1a, right). Because the Marcus expression contains three unknown parameters (ΔG_0_, λ, and H_ab_) and we have only two variables (*k*_nr_ and T), additional information must be obtained for one of the three Marcus terms. Density functional theory (DFT) is used for calculating ΔG_0_, which corresponds to the difference in zero-point free energy between the structurally relaxed excited state and the ground state.^37^ Values for λ and H_ab_ can then be determined from the fits of the experimental time-resolved absorption data and thereby provide complementary diagnostics for assessing the nature of the MC excited states. In Fe­(II) polypyridyl complexes, the ^5^T_2_ excited states typically exhibit large reorganization energies (λ ≈ 10,000 – 14,000 cm^–1^)^37^ due to the population of two electrons in the antibonding e_g_* orbitals, which results in a ∼ 10% increase in the metal–ligand (M-L) bond length and a 20 – 25% increase in molecular volume as compared to the ground state (Figure 1b, left). This large structural change during GSR translates to a large reorganization energy barrier for this process.^2^ In contrast, we have observed that corresponding Co­(III) analogues show substantially smaller reorganization energies (λ ≈ 5,000 – 7,000 cm^–1^),^2^ a nearly 50% reduction that is consistent with an assignment of the ^3^T_1_ ligand-field state as the lowest-energy excited state, in which only one electron occupies the antibonding e_g_* level (Figure 1b, right).^33,50^

Reorganization energy alone, however, is insufficient for a definitive spin-state assignment because variations in ligand structure and flexibility can modulate structural distortions, which in turn will be reflected in the magnitude of λ. Therefore, the electronic coupling parameter H_ab_ provides an additional diagnostic. Relaxation from the ^5^T_2_ excited state of Fe­(II) to the ^1^A_1_ ground state requires a spin change of two (i.e., S = 2 for the ^5^T_2_ state to S = 0 for the ^1^A_1_ state). No matrix element can directly couple two electronic states that differ by two spin quanta, meaning that direct coupling between these states does not occur. One can construct a viable coupling pathway by envisioning two ΔS = 1 interactions, specifically, quantum mechanical mixing of the ^5^T_2_ state with the lowest energy (thermally inaccessible) ^3^T_1_ state, which in turn couples with the ^1^A_1_ ground state. The overall second-order interaction is expected to be weak, consistent with the small values of H_ab_ (∼4 to 6 cm^–1^) derived from experiment.^37^ In contrast, values of H_ab_ obtained for Co­(III) polypyridyl complexes as well as Fe­(II) compounds with strong-field *N*-heterocyclic carbene (NHC) ligands are nearly an order of magnitude larger (16 and 35 cm^–1^),^2^ suggesting a much stronger electronic interaction than can be easily rationalized through a direct-coupling mechanism. Moreover, an inverse relationship between ΔG_0_ and H_ab_ is observed in the case of Co­(III) polypyridyls, a result expected from perturbation theory for two states that are directly coupled: both of these observations are wholly consistent with the ^3^T_1_ state as the lowest energy excited state of this system.^2,32^

Additional support for spin-state assignment can be obtained from a transition state theory analysis of the activation entropy (ΔS^‡^) obtained from the intercept of the linearized Eyring equation by plotting ln­(*k*_nr_/T) versus 1/T (Figure 1a, center). Fe­(II) chromophores with ^5^T_2_ excited states, particularly spin-crossover complexes where the identity of the lowest energy excited state is unambiguous, are characterized by ΔS^‡^ values ranging from – 5 to – 6 cm^–1^ K^–1^.^51^ The negative value reflects the reduction in both molecular volume and magnitude of the vibrational partition function associated with conversion from a high-spin to a low-spin state in an isoelectronic system.^37^ In contrast, Co­(III) polypyridyl and Fe­(II)-NHC complexes alluded to above display smaller values of ΔS^‡^ (−3 to – 4 cm^–1^ K^–1^),^2^ indicating a reduction in the magnitude of the structural change associated with GSR in these systems. This is qualitatively similar to the changes documented earlier with regard to the Marcus reorganization energies and creates a self-consistent picture of the difference in the nature of the lowest energy excited state in these classes of chromophores.^2^

Collectively, λ, H_ab_, and ΔS^‡^ therefore provide complementary diagnostics for identifying the spin and electronic character of MC excited states. As we will discuss next, the differences identified among these various classes of isoelectronic first-row metal complexes have a profound impact on the ability of these chromophores to act as catalysts for photoredox reactions.

## Spin-State and Reorganization Dynamics in MC Photochemistry

Since ligand-field excited states involve redistribution of electrons within the metal-centered d-orbitals, photoinduced electron transfer proceeds along reaction coordinates that differ fundamentally from CT-state photochemistry and can involve competing pathways with different spin states and equilibrium geometries. Consequently, evaluating productive photoinduced electron transfer from ligand-field excited states requires considering not only the thermodynamic driving force but also the spin requirements and structural reorganization.^1^

When considering Co­(III) polypyridyl chromophores, the lowest-energy excited state corresponds to a ^3^T_1_ MC state with an electronic configuration of (t_2g_)^5^(e_g_*)^1^. Because Co­(IV) species are poorly stabilized under low-to-moderate ligand-field conditions, oxidative quenching is expected to be highly endothermic. In contrast, reductive quenching readily generates a stable high-spin Co­(II) species with a (t_2g_)^5^(e_g_*)^2^ configuration, together with a substrate radical cation. In principle, two mechanistic pathways can lead to this product: (1) electron transfer into an e_g_* orbital, which directly forms the final high-spin Co­(II) product (Figure 2a, yellow box), or (2) electron transfer into a lower-lying t_2g_ orbital, initially generating a low-spin Co­(II) species that subsequently converts to the high-spin configuration (Figure 2a, gray box). In both cases, exchange interactions between the Co­(II) center and the substrate radical generate two possible total spin states (S_T_ = 1 and 2 for path 1, and S_T_ = 0 and 1 for path 2). Therefore, a reaction trajectory that conserves spin can proceed through the S_T_ = 1 state, which is accessible in both scenarios; hence, both mechanistic pathways are viable.

**Figure 2:**
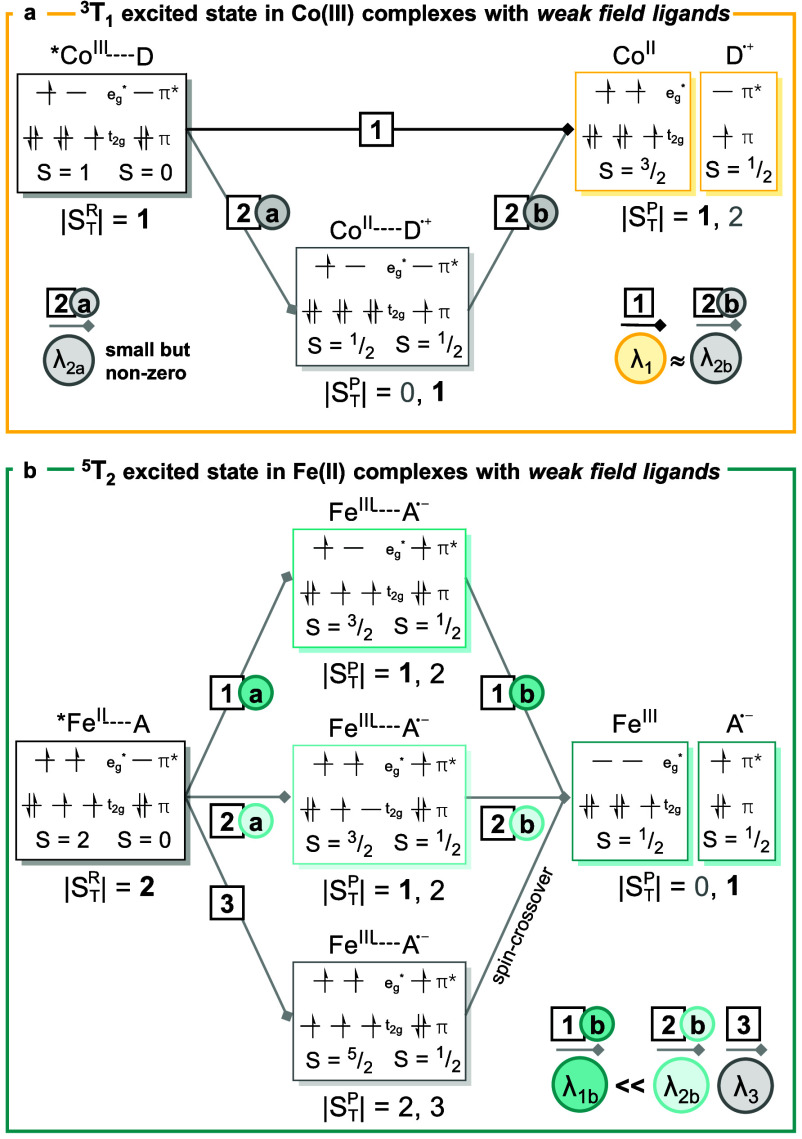
Simplified representation of spin-selection considerations for photoinduced electron transfer, where the total spin of the reactant (|S_T_^R^|) and product (|S_T_^P^|) states are determined from the individual microspin states (S) of the metal complex and the donor or the acceptor. (a) Electron transfer from a donor (D) to the ^3^T_1_ excited state of a Co­(III) complex. (b) Electron transfer from the ^5^T_2_ excited state of an Fe­(II) complex to an electron acceptor (A).

The inner-sphere reorganization energy (λ_i_) of this process is mainly determined by the addition of a second electron to the σ-antibonding e_g_* orbitals, which occur along both pathways (Figure 2a, path 1a and 2b). Path 2 includes an additional step involving electron population of the t_2g_ orbitals before the spin flip, which could slightly increase λ_i_. However, because π-interactions between polypyridyl ligands and first-row transition metals are generally weak, this difference is expected to be small. A more important distinction is that pathway 2 initially forms an excited electronic state of the Co­(II) complex, which likely makes pathway 1 the more thermodynamically favorable route. In either case, electron transfer from the ^3^T_1_ excited state follows a relatively simple reaction coordinate dominated by population of antibonding orbitals in the reduced Co­(II) complex. As a result, the inner-sphere reorganization energy is comparatively modest, allowing efficient photoinduced electron transfer associated with the MC excited state.

This picture changes dramatically for Fe­(II) polypyridyl complexes, which typically populate a ^5^T_2_ MC excited state with a (t_2g_)^4^(e_g_*)^2^ configuration.^25^ Much like oxidation of Co­(III) to Co­(IV), reduction to Fe­(I) is not thermodynamically viable for the ligand environments considered here. Instead, Fe­(II)-based chromophores undergo oxidative quenching, forming a low-spin Fe­(III) species with an electronic configuration of (t_2g_)^5^(e_g_*)^0^ together with a substrate radical anion.^52^ However, electron transfer from the ^5^T_2_ excited state initially generates Fe­(III) spin states that do not correspond to the thermodynamically preferred low-spin ground state and must subsequently relax to the low-spin Fe­(III) state (Figure 2b). As a result, electron transfer from the ^5^T_2_ excited state involves a significantly more complex reaction coordinate than in the Co­(III) case, leading to large inner-sphere reorganization energies (10,000 – 12,000 cm^–1^) due to changes in population of the σ-antibonding orbital and the accompanying spin-state change. These considerations also affect the thermodynamic analysis of Fe­(II) photoredox chemistry. The commonly applied Rehm–Weller formalism assumes that electron transfer directly produces the Fe­(III) ground state.^53^ However, electron transfer from the ^5^T_2_ excited state first forms higher-energy Fe­(III) intermediates, reducing the effective excited-state oxidation potential and leading to a significant overestimation of the redox power of Fe­(II)-based photocatalysts.^54^

In considering the spin-state evolution and reorganization energy required to proceed to product formation in photoredox reactions containing ^5^T_2_ or ^3^T_1_ MC excited states, our analysis suggests that photosensitizers with ^5^T_2_ states are unlikely to undergo productive redox chemistry due to the population of intermediate spin states, which require excess reorganization energy to form the low-spin Fe­(III) products. In contrast, ^3^T_1_ excited states provide a direct, spin-allowed pathway for the formation of high-spin or low-spin reduced photocatalysts and are associated with significantly smaller reorganization energies, which should promote favorable reactivity.

## Reactivity Constraints in ^5^T_2_ MC Excited States

As discussed above, electron transfer from ^5^T_2_ MC excited states is expected to be strongly constrained by spin-state requirements and large structural reorganization energies,^1^ which can significantly suppress productive electron transfer. However, several studies have reported photoredox reactivity from ^5^T_2_ MC excited states in Fe­(II) polypyridyl complexes, but the mechanistic origin of this reactivity has remained unclear.^55,56^ This has motivated us to directly investigate the feasibility of electron transfer from the ^5^T_2_ excited state using transient absorption spectroscopy.

We investigated the oxidative quenching of the Fe­(II)-based ^5^T_2_ state (Figure 3a) by 2,3-dichloro-5,6-dicyano-1,4-benzoquinone (DDQ) with *E*_red_ = 0.14 V (vs Fc/Fc^+^) in acetonitrile.^57^ As photocatalysts, we selected Fe­(II) polypyridyl complexes containing 2,2′-bipyridine (bpy) and 2,2′:6′,2″-terpyridine (terpy) ligands. Using their ground-state redox potentials together with stored excited-state energies of 0.7–1.0 eV,^37^ the Rehm–Weller approximation^53^ predicts excited-state oxidation potentials of approximately – 0.16 to – 0.26 V vs Fc/Fc^+^, corresponding to estimated driving forces of – 0.3 to – 0.4 eV for reduction of DDQ (Figure 3b). However, despite DDQ concentrations of up to 250 mM, excited-state lifetimes as long as 22 ns, and a thermodynamic driving force of approximately – 0.32 eV, no excited-state quenching was observed (Figure 3c). In principle, these conditions should enable diffusion-controlled electron transfer and yield at least 95% quenching.^58^ The absence of measurable quenching therefore places an upper limit of ∼ 1 × 10^7^ M^–1^ s^–1^ on the electron-transfer rate constant, indicating strongly kinetically inhibited electron transfer from the ^5^T_2_ excited state.

**Figure 3:**
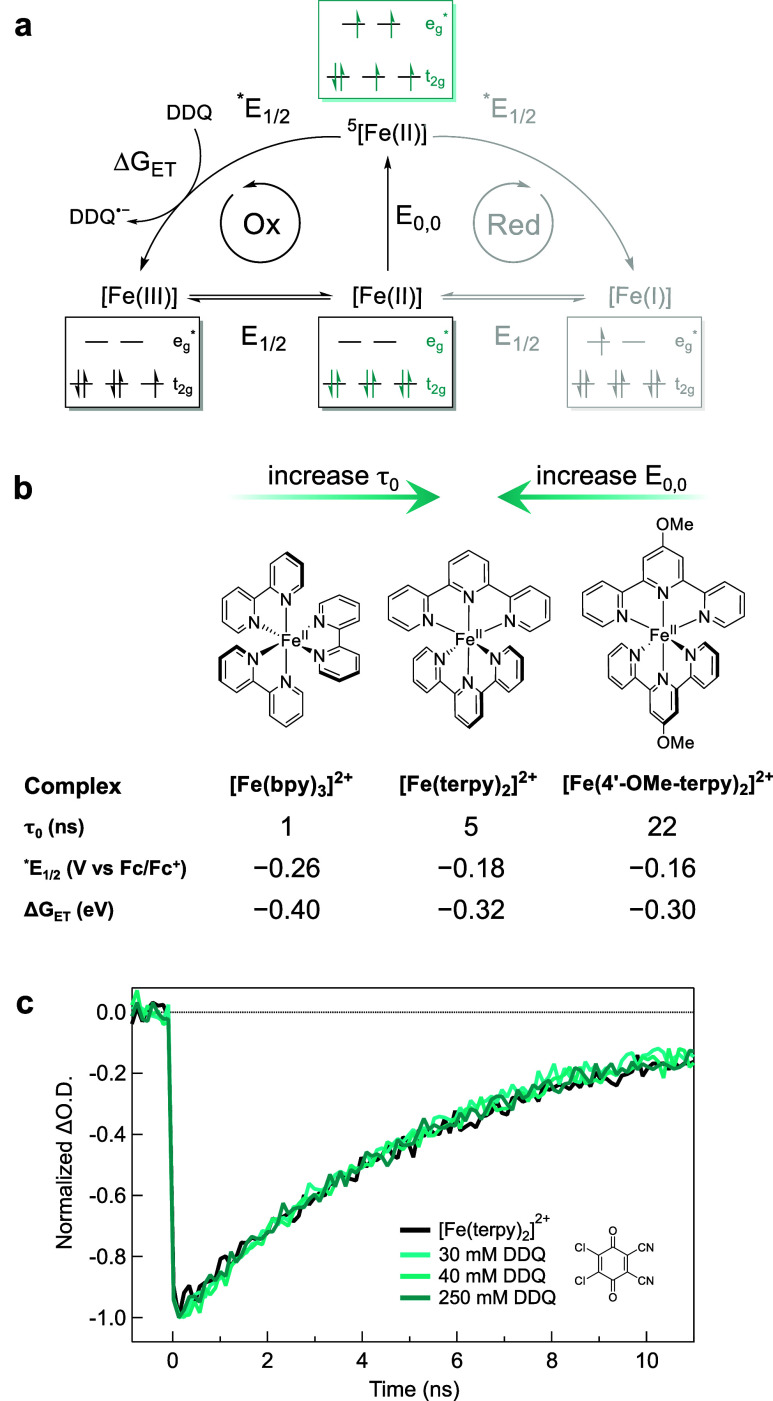
(a) Latimer diagram for Fe­(II) polypyridyl chromophores. (b) Fe­(II)-polypyridyl complexes with corresponding excited-state lifetime (τ_0_), the excited-state oxidation potential (*E_1/2_), and the driving force for electron transfer to DDQ (ΔG_ET_). (c) Single wavelength transient absorption measurement of [Fe­(terpy)_2_]^2+^ in the presence of 0 to 250 mM DDQ.

While electron transfer cannot be completely excluded, these results are consistent with the notion that spin-state restrictions and large reorganization-energy barriers strongly suppress reactivity from the ^5^T_2_ excited state, despite apparently favorable thermodynamic and kinetic conditions.

## MC Excited-State Photoreactivity in CobalT(III) Polypyridyl ComplexeS

As established above, the photoreactivity of Fe­(II) polypyridyl complexes is severely limited by the electronic and structural constraints associated with their lowest-energy ^5^T_2_ MC excited state.^1^ In contrast, the relatively higher-energy ^3^T_1_ MC excited state is a more potent redox agent while also benefiting from reduced reorganization penalties and relaxed spin constraints. Both factors should conspire toward the realization of a useful, earth-abundant chromophore for metal-centered photoredox catalysis.

Similar to the Fe­(II) ^5^T_2_ state, we evaluated the electron-transfer reactivity of the Co­(III) ^3^T_1_ excited state using Stern–Volmer quenching studies.^4^ Because these complexes are nonemissive, the excited-state energy was estimated from the ∼ 0.4 eV increase in ligand-field strength relative to Fe­(II) tris-bipyridyl complexes (0.9 eV),^59^ giving an estimated stored energy of ∼ 1.3 eV for [Co­(bpy)_3_]^3+^. Combined with the ground-state reduction potential (∼−0.05 V vs Fc/Fc^+^), this corresponds to an estimated excited-state reduction potential of ∼ 1.25 V vs Fc/Fc^+^.^53^ Stern–Volmer studies using [Co­(4,4′-Br_2_bpy)_3_]^3+^ (where 4,4′-Br_2_bpy = 4,4′-dibromo-2,2′-bipyridine) and a series of electron-rich arenes (0.9–1.3 V vs Fc/Fc^+^).^4^

This value is substantially higher than those of widely used photocatalysts such as [Ru­(bpy)_3_]^2+^ (*E*_red_* = 0.32 V vs Fc/Fc^+^)^60^ and [Ir­(dF­(CF_3_)­ppy)_2_(dtbbpy)]^+^ (*E*_red_* = 0.81 V vs Fc/Fc^+^),^61^ both of which operate from CT excited states. Combined with an excited-state lifetime on the nanosecond time scale (sufficient to support diffusion-controlled bimolecular reactivity), these results demonstrate that Co­(III) polypyridyl complexes should enable transformations that are inaccessible to conventional Ru­(II)- and Ir­(III)-based photocatalysts. In a collaborative effort with the MacMillan group,^3^ it was found that [Co­(4,4′-Br_2_bpy)_3_]^3+^ can indeed promote the photocatalytic C­(sp^2^)–N coupling of aryl amides with aryl boronic acids (Figure 4b, left),^3^ a transformation of considerable interest for the synthesis of pharmaceutically relevant molecules.

**Figure 4:**
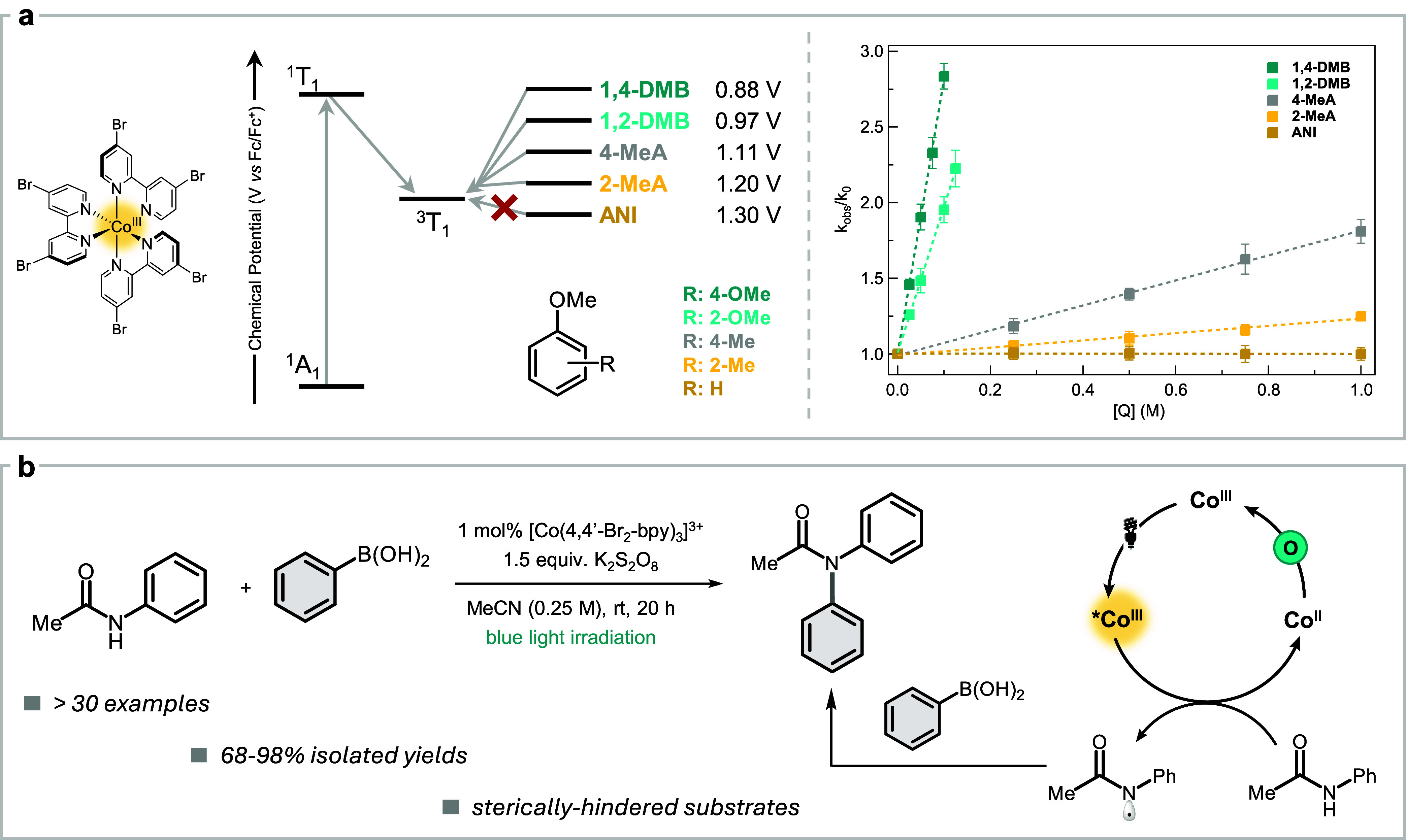
(a) Chemical structure of [Co­(4,4′-Br_2_bpy)_3_]^3+^(left), simplified energy diagram depicting photoinduced electron transfer from the ^3^T_1_ MC excited state to different benzene derivatives (center), and the resulting Stern–Volmer analysis of bimolecular quenching kinetics between [Co­(4,4′-Br_2_-bpy)_3_]^3+^ and the electron donors (right). (b) Photocatalytic C­(sp^2^)–N coupling of aryl amide and aryl boronic acid facilitated by [Co­(4,4′-Br_2_-bpy)_3_]^3+^and the proposed catalytic cycle (right).

Mechanistic investigations revealed a near thermoneutral electron transfer process from [Co­(4,4′-Br_2_-bpy)_3_]^3+^ to acetanilide (*E*_ox_ ≈ 1.27 V vs Fc/Fc^+^) followed by oxidation with K_2_S_2_O_8_ to regenerate the Co­(III) photocatalyst and close the catalytic cycle.

Guided by these mechanistic insights and the limitations of traditional metal-mediated cross-coupling methods, reaction scope studies revealed broad functional-group tolerance and high efficiency, including sterically hindered ortho- and diortho-substituted boronic acids.^62,63^ In contrast, conventional Ru­(II), Ir­(III), and organic photocatalysts delivered yields below 10% under analogous conditions, compared to >90% with Co­(III) photocatalysts. We believe these results highlight the enormous potential of MC excited states in Co­(III) polypyridyl complexes to facilitate highly oxidizing and mechanistically distinct photochemistry that dwarfs the capabilities of conventional charge-transfer photocatalysts under comparable conditions.

## An Unexpected Benefit: The Marcus Inverted Region Behavior of Cobalt(III)

To rationalize the exceptional photoredox performance of these Co­(III) polypyridyl complexes, we investigated how the ^3^T_1_ MC excited state supports efficient bimolecular reactivity. Despite a stored energy of ∼ 1.3 eV, lifetimes of several nanoseconds enable efficient excited-state quenching, unlike most high-energy MC excited states.^64^

Marcus theory again provides a powerful quantitative framework linking excited-state decay rates to the ΔG_0_ and λ. In systems where structural reorganization dominates (λ > |ΔG_0_|), relaxation occurs in the Marcus normal region, where increasing excited-state energy accelerates nonradiative decay. This regime is characteristic of Fe­(II) polypyridyl complexes, where population of the ^5^T_2_ state involves promotion of two electrons into the antibonding e_g_* orbitals, leading to large structural distortions, substantial λ, and rapid excited-state relaxation.^37^ Consequently, enhancing ΔG_0_ in such systems often shortens excited-state lifetime, fundamentally limiting bimolecular photoredox efficiency.^14,65^

In contrast, operation in the Marcus inverted region (λ < |ΔG_0_|) is particularly advantageous for photoredox applications because higher excited-state energies lead to an increase in the excited-state lifetime. On this basis, we hypothesized that the GSR dynamics associated with the ^3^T_1_ MC excited state in Co­(III) polypyridyl complexes operate in the Marcus inverted region. Unlike Fe­(II) analogues, the ^3^T_1_ MC state involves population of only a single antibonding e_g_* orbital, reducing structural distortions and lowering λ. In addition, the higher metal-centered charge of Co­(III) increases the ligand-field splitting relative to Fe­(II), further stabilizing the metal-centered excited state and supporting operation in the Marcus inverted region.

Experimental support for this hypothesis was obtained from a series of Co­(III) complexes containing substituted bipyridyl ligands, which exhibit comparable structural reorganization energies (Figure 5).^2,3^ Across this series, systematic variation of ligand-field strength modulated the stored excited-state energy from approximately 1.25 to 1.35 eV, while the excited-state lifetime increased from 0.47 to 6.5 ns.^3^ The resulting Marcus analysis yields a reorganization energy of approximately 4500 cm^–1^ (∼0.55 eV), confirming inverted-region behavior.^3^ This provides a mechanistic basis for the remarkable photoredox activity of Co­(III) complexes containing ^3^T_1_ MC excited states, where both thermodynamic driving force and the probability of bimolecular encounters increase simultaneously.^12^ Therefore, operation in the Marcus inverted region emerges as a key design principle for enabling efficient photochemistry from short-lived, metal-centered excited states.

**Figure 5:**
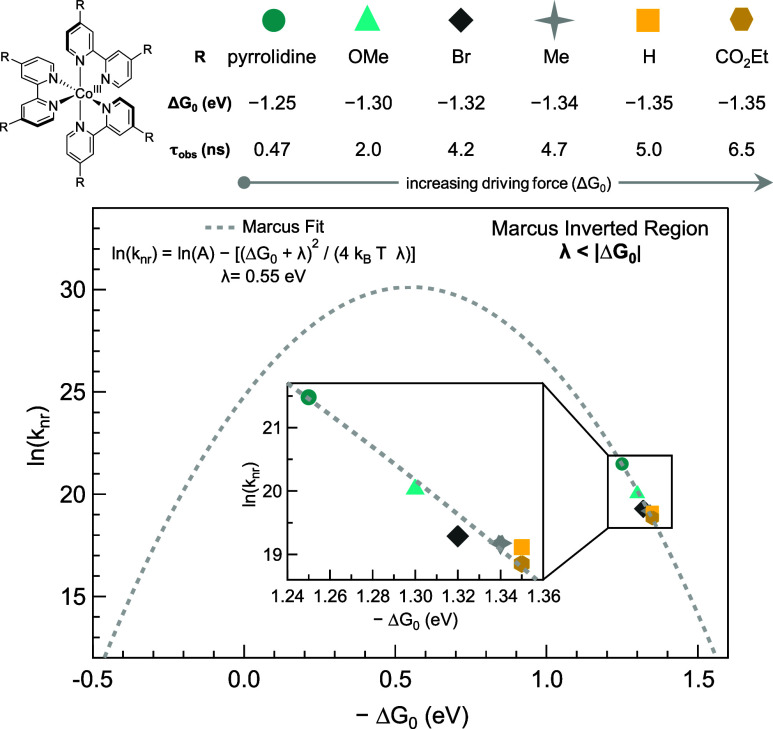
Plot of experimentally determined rate constants (*k*_nr_) as a function of driving force (−ΔG_0_) for ground-state recovery in Co­(III) polypyridyl complexes. The data are described by Marcus theory with a reorganization energy (λ) of 0.55 eV.

## Controlling MC Photoreactivity through Ligand Field Modulation

To guide the design of Fe­(II) and Co­(III)-based photocatalysts, it is essential to consider how ligand-field strength influences the spin state of MC excited states. In Fe­(II) complexes, increasing the ligand field strength destabilizes the ^5^T_2_ excited state, allowing the ^3^T_1_ state to become the lowest energy MC excited state.^28,29,31,38,66–70^ This change in excited-state character reduces spin restrictions and λ barriers for electron transfer, thereby mirroring the behavior observed from the ^3^T_1_ excited states of Co­(III) complexes but acting as a reductant as opposed to an oxidant. The ^3^T_1_ excited state of Fe­(II) with a (t_2g_)^5^(e_g_*)^1^ configuration can donate an electron from the e_g_* orbital to the substrate π* orbital (Figure 6a), directly generating a low-spin Fe­(III) complex with a (t_2g_)^5^(e_g_*)^0^ configuration, along with a substrate radical anion. As the electron transfer primarily involves depopulation of a single antibonding orbital, the associated reorganization energy should be significantly attenuated relative to the same process arising out of the ^5^T_2_ excited state. Thus, with appropriate photosensitizer design, Fe­(II) complexes featuring ^3^T_1_ MC excited states can be expected to engage in productive photoredox chemistry under favorable thermodynamic conditions.

**Figure 6:**
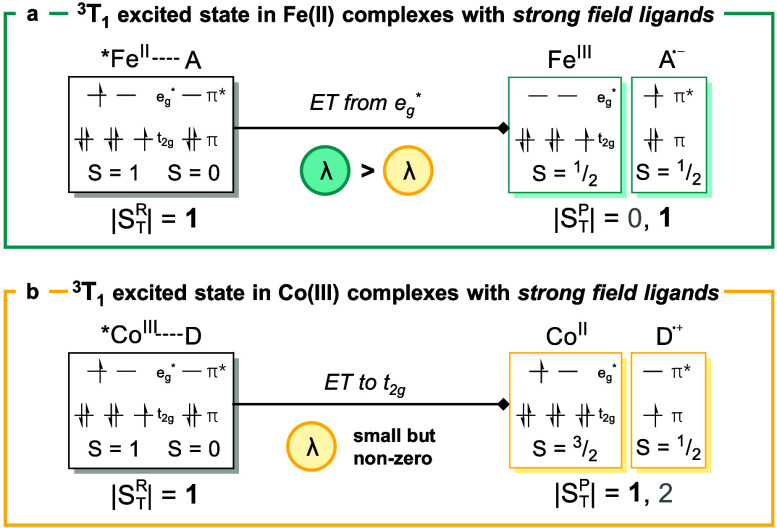
(a) Simplified schematic of spin selection rules (and qualitative reorganization energy considerations) for photoinduced electron transfer to an electron acceptor (A) from the ^3^T_1_ excited state of Fe­(II) complexes. (b) Analogous schematic for photoinduced electron transfer from a donor (D) to the ^3^T_1_ excited state of Co­(III) complexes.

In Co­(III) complexes, access to ^3^T_1_ excited states similarly presents an opportunity to minimize reorganization energy during electron transfer. In contrast to Fe­(II), reduction of a Co­(III) excited state will lead to high-spin products in systems with weak-to-moderate field ligands. This gives rise to significant geometric and electronic reorganization (though not prohibitive), represents an energetic cost in the overall catalytic process. To address this issue, we propose that the use of strong-field ligands sufficient to stabilize Co­(II) in a low-spin (^2^E) ground-state configuration represents an intriguing approach for enabling a more efficient reductive pathway (Figure 6b). Here, electron transfer from an electron donor to the Co­(III) ^3^T_1_ excited state populates the t_2g_ orbital, directly forming a low-spin Co­(II) species. Because this process involves the addition of an electron into a largely nonbonding t_2g_ orbital, the associated structural reorganization is minimized. Consequently, the reorganization energy is expected to be small, facilitating more efficient electron transfer than pathways that produce high-spin Co­(II) species, requiring population of antibonding e_g_* orbitals.

## Selectivity of Ligand-Field Excited States

In addition to considerations of spin-state (and concomitant reorganization energies), enhanced selectivity is an added potential benefit when leveraging ligand-field excited states for applications in photoredox. There are three major classes of excited states that have been utilized for photoredox: (1) charge-transfer excited states (typically inorganic but some organic chromophores, as well),^71^ (2) π-π* excited states (primarily organic-based sensitizers),^8^ and (3) metal-centered excited states of the kind that have been discussed in this Account.^14,72,73^ In all three of these cases, exergonicity of the reaction is obviously a necessary criterion to satisfy, but the specific nature of these states impacts how selective they are regarding oxidative versus reductive chemistry. In the case of MLCT states (e.g., the ^3^MLCT of [Ru­(bpy)_3_]^2+^), the excited state possesses both an oxidant and a reductant (Ru­(III) and bpy^.‑^, respectively). The reducing equivalent is on the periphery of the molecule: in a bimolecular reaction where both oxidation and reduction of a substrate are thermodynamically viable, MLCT states will be preferentially reductive in nature. LMCT excited states reverse this condition, wherein the oxidizing equivalent (e.g., phtmeimb^.+^ for the ^2^LMCT of [Fe­(phtmeimb)_2_]^+^)^74^ will be the moiety that defines the initial encounter with the substrate. Absent specific synthetic design, these sorts of spatial considerations are typically less prominent for organic π-π* excited states: both the hole and electron (i.e., π^+^ and π^–^) are equally accessible so the chemistry will be dominated by thermodynamics considerations. In contrast, MC excited states are wholly localized on the metal. While this can present challenges insofar as the substrate needs to gain sufficient access to the metal center to react, it also means that the primary coordination sphere (i.e., the ligand architecture) can be leveraged to control this interaction. Efforts along these lines are currently underway.

Finally, there is an intrinsic selectivity associated with metal-centered excited states that can be turned to an advantage in certain cases. Consider the d^6^ metals that have been the subject of this Account. [Fe­(bpy)_3_]^2+^ and [Co­(bpy)_3_]^3+^ are isoelectronic and are characterized by identical manifolds of ligand-field excited states (albeit with a different energetic profile). Regardless of the identity of the photoactive MC state, these two isoelectronic compounds are likely to undergo orthogonal chemistries. Unlike MLCT or LMCT, the formal oxidation state of the metal in an MC state is unchanged from the ground state: the Co center of the ^3^T_1_ state is still Co­(III), and the ^5^T_2_ (or ^3^T_1_) state of an iron-based chromophore is still Fe­(II). This creates restrictions in terms of the type of thermodynamically viable reactivity one can expect to observe. As highlighted in Figure 2b, oxidation of an iron-based MC excited state will yield low-spin Fe­(III). [Fe­(bpy)_3_]^2+^ (as is the case with most Fe­(II) polypyridyls) exhibits an electrochemically reversible Fe­(II)/Fe­(III) oxidation in its cyclic voltammetry,^75^ making reduction of a substrate from an Fe­(II)-based MC state thermodynamically feasible. The corresponding oxidative reaction would lead to reduction of Fe­(II) to Fe­(I), an oxidation state that cannot be accessed at reasonable potentials in these types of compounds (indeed, reduction of bpy to bpy^−^ occurs at far less negative potentials). This situation is reversed for Co­(III): data described earlier in this Account shows that the ^3^T_1_ excited state of this class of chromophores is potent oxidant, yielding a stable Co­(II) product. Oxidation of the ^3^T_1_ state requires formation of a Co­(IV) species, an oxidation state that is difficult to access absent the formation of a terminal oxo species.^76^ Thus, despite being isoelectronic, the nature of redox chemistry associated with ligand-field excited states drives Fe­(II) and Co­(III) to carry out fundamentally different reactions. A range of situations – including some where both oxidative and reductive chemistries can be viable (e.g., Ni­(II)^73,77–79^ ) – can be found across the first transition series and represents an additional layer of selectivity that is difficult to reproduce in either charge-transfer or π-π*-based excited states.

These insights underscore ligand-field modulation as a strategy to overcome the intrinsic spin and reorganization challenges of first-row transition-metal photocatalysts, while enabling unique reaction selectivity, providing a promising route toward the development of sustainable, earth-abundant alternatives to noble metal-based photocatalysts.

## Concluding Remarks

Early strategies for designing first-row transition metal complexes that mimic their second- and third-row counterparts largely focused on reproducing long-lived and highly energetic charge-transfer states. In contrast, the ^3^T_1_ MC excited state, typically accessed in Co­(III) systems, have emerged as promising platforms for selective photoredox chemistry, exhibiting success with C­(sp^2^)–N bond formation,^3^ that are inaccessible to the widely employed Ru­(II)- and Ir­(III)-based photocatalysts.

However, two major challenges remain for Co­(III) based photocatalysts: weak visible-light absorption and substantial reorganization energies following electron transfer. The first arises from the Laporte-forbidden ligand-field transitions. Strategies such as symmetry breaking and ligand design incorporating π-extension^80–82^ represent promising approaches to enhance absorption cross sections, although improvements in catalytic performance have remained limited. The second challenge is minimizing reorganization energy during electron transfer; Hence stabilization of low-spin Co­(II) through strong-field ligands appears to be a particularly important design principle for Co­(III)-based chromophores and potentially other first-row transition-metal systems.

In the case of Fe­(II) complexes, strengthening the ligand field can stabilize the more reactive ^3^T_1_ manifold, but typically at the expense of shortening excited-state lifetimes. An alternative strategy that emerged from our work involves intercepting higher-lying charge-transfer states before relaxation into the metal-centered manifold,^6,83^ similar to hot-electron injection in dye-sensitized solar cells.^10,84,85^ Accessing these nonequilibrated higher-energy states enables photoredox reactivity with substantially enhanced redox potentials, which opens a new direction in photochemistry; however, realizing the full potential of this strategy will require improved control over excited-state relaxation pathways, including slowing internal conversion and intersystem crossing, as well as a deeper understanding of preassociation and its role in enabling ultrafast electron transfer.

In summary, we believe that our recent work has demonstrated that, rather than focusing solely on efforts to make first-row transition-metal complexes mimic second- and third-row congeners, embracing the unique reactivity of MC excited states can transform what were perceived as intrinsic limitations into opportunities for fundamentally new photochemical pathways. By controlling excited-state energetics, spin state, structure, and excited-state dynamics, these systems can access reactivity patterns distinct from conventional charge transfer-based chromophores opening new directions for sustainable photoredox catalysis based on earth-abundant photocatalysts.
